# Impact of Covid-19 on the Behavior of Community Residents With Suspected Transient Ischemic Attack

**DOI:** 10.3389/fneur.2020.590406

**Published:** 2020-10-16

**Authors:** Sichen Yao, Beiru Lin, Yang Liu, Yunhe Luo, Qianqian Xu, Jiating Huang, Zhimin Yan, Xiaochuan Liu

**Affiliations:** ^1^Wujing Community Health Service Center, Shanghai, China; ^2^Maqiao Community Health Service Center, Shanghai, China; ^3^Department of Neurology, Minhang Hospital, Fudan University, Shanghai, China; ^4^WanNan Medical College, Anhui, China

**Keywords:** COVID-19, community, TIA = transient ischemic attack, stroke, public awareness

## Abstract

**Background and Purpose:** To investigate the impact of the novel coronavirus disease 2019 (Covid-19) on the behavior of those seeking medical attention for community residents suspected of having had a transient ischemic attack (TIA) during the pandemic.

**Methods:** This was a community-based cross-sectional study with residents living in two communities located in the suburb of Shanghai. A face-to-face interview was prospectively conducted from 20 May 2020 to 30 June 2020 between community physicians and the community residents. Suspected TIA that occurred during the pandemic was identified by symptoms recalled from the community residents. The behavior of seeking medical attention after the suspected TIA was investigated.

**Results:** A total of 873 community residents (517 from the Wujing community and 356 from the Maqiao community) took part in face-to-face interviews. Among them, 143 (16.38%) suspected TIA cases were identified. Less than 20% of the community residents suspected of having a TIA went to the hospital during the Covid-19 pandemic. The most common reason for not seeking medical care during the pandemic was still that symptoms disappeared quickly (94.9%); however, the pandemic did have an impact on behavior. Fear of in-hospital infection (55.1%) and of complicated procedures involved in seeking medical attention during the pandemic (55.9%) made community residents hesitate to seek medical attention after the suspected TIA. Residents with a dual attack within 1 week or with aphasia or dysarthria were more likely to seek medical attention during the pandemic.

**Conclusion:** Our study indicated that the Covid-19 pandemic negatively affected the behavior of those seeking medical attention among community residents with suspected TIA and this might explain part of the reduction in patients presenting with stroke or TIA observed from other reports.

## Introduction

The novel coronavirus (COVID-19) has posed a great challenge to the global health care system, especially for those less developed countries (1, 2). With the reallocation of health resources to support the treatment of patients with Covid-19, negative outcomes were observed among other departments of the health care system. Stroke specialists worldwide have reported seeing a significant drop in the number of patients presenting at the stroke center with stroke or transient ischemic attack (TIA) (3–7). Speculation about the cause of this decline include fear of in-hospital infection, the negative impact of the stay-at-home order, and the potential decline in stroke incidence during the pandemic. It's worth noting that several studies from different countries have reported a significant reduction in presentation with TIA or minor stroke, while the risk of recurrent stroke of these patients was not less than that of major stroke (4–6). To our knowledge, only limited data have been available to explore the impact of the Covid-19 pandemic on the behavior of those community residents with suspected TIA seeking medical care. In other words, the pandemic had played a role in the community residents' reluctance to seek medical care after the TIA. In this study, we conducted face-to-face interviews with community residents to identify those who had suspected TIA during the pandemic and investigate the actions they took after the symptom onset. Factors associated with those seeking medical attention immediately after the suspected TIA were also explored.

## Methods and Materials

### Study Design

This study was a community-based cross-sectional survey through face-to-face interviews between community physicians and community residents who seek medical help at community health service centers. Community health service centers in our country have played a role in the management of primary health care and secondary prevention of diseases. Community residents usually go to the community health service centers for the management of chronic diseases such as hypertension or diabetes. Community physicians, also known as general practitioners with 3 years of general practitioner training, treat acute and chronic illnesses and also promote health education to the community residents. The face-to-face interviews were conducted at two community health service centers located in the suburbs of the Minhang district, Shanghai. Wujing community health service center serves about 90,000 residents living in the Wujing community. Maqiao community health service center manages the primary health care for about 100,000 residents living in the Maqiao community. The direct distance between these two health service centers was about 12 km. This study protocol was approved by the Review Board of each health service center. Written informed consent was obtained from all participants. The original data that support this finding can be obtained from the corresponding author upon reasonable request.

### Data Collection and Risk Factor Definition

Data were prospectively collected from May 20, 2020 to June 30, 2020. Qualified community physicians with 5 years' working experience conducted the face-to-face interviews to collect the residents' information, including demographic data, living habits, medical history, suspected TIA symptoms they experienced during the pandemic (from 1 February 2020 to 30 April 2020), and the action they took after the symptom onset. The details of the survey were shown in the Supplementary Material. We excluded residents who refused to participate at the time they were told the purpose of the survey. Residents with difficulties in communicating with the interviewers (such as dementia or impaired hearing) were also excluded. As for residents who seek medical help at the community health service center accompanied by their family members, we would also have a talk with them during face-face interviews to confirm the symptoms. All the community physicians (Sichen Yao, Beiru Lin) accepted nearly 1 h of online stroke education before the program started from an experienced stroke specialist (Zhimin Yan) to help them identify the transient ischemic attack symptoms and interpret the symptoms in a way that are understandable for community residents. Physical examinations had also been conducted during face-to-face interviews to rule out other common etiologies that may mislead the diagnosis of TIA.

The smoking and drinking habits of community residents were self-reported. Past medical history (hypertension, diabetes, hyperlipidemia, cancer, previous stroke) was defined according to standard definitions and with a medical record from local tertiary hospitals. As for residents with hypertension, an additional question regarding the control of the blood pressure during the pandemic would be asked. Blood pressure higher than 140/90 mmHg was marked in the survey even for only one instance.

### Suspected TIA Cases and Public Knowledge of TIA

During the pandemic, suspected TIA cases were identified by symptoms recalled from community residents through face-to-face interviews conducted by trained community physicians. Residents with the following symptoms were considered as suspected TIA cases: sudden motor weakness or sensory deficit in two limbs or one limb and the face; sudden dysphasia; and sudden monocular or binocular blackening, blurred vision, and sudden blackening or absence of visual field (8). As for the suspected TIA cases, the duration of the symptoms and whether there is a “dual” suspected TIA within seven days were also recorded. Of those suspected TIA cases who didn't seek medical help, we listed three predefined potential reasons for them to choose, which included (1) the symptoms disappeared quickly, (2) fear of in-hospital infection with Covid-19, and (3) the complicated nature of procedures for seeking medical help during the pandemic hindered them from going to the hospital. In the last case, we investigated community residents' knowledge of the meaning of the term “transient ischemic attack.” They were considered to know about TIA if they could name at least one of the typical symptoms and knew that it is related to ischemic stroke.

### Statistical Analysis

Baseline characteristics were compared by chi-square tests or Fisher exact tests for categorical variables and two-sample *t*-tests for continuous variables. The stroke risk prediction scores ABCD2 and ABCD3 for TIA were calculated by adding the score corresponding to the predictors (age, blood pressure, clinical signs, duration of the symptoms, diabetes, and dual attack within 7 days) (9). Factors independently associated with seeking medical help after the suspected TIA were identified through multivariate logistic regression analysis after adjusting for eight confounders (age, gender, drinking habits, smoking habits, hypertension, diabetes, previous stroke, and cancer of any type). Statistical analysis was performed on SPSS version 25.0 (IBM Corp., New York). A two-tailed *P*-value < 0.05 was considered statistically significant.

## Results

### The Baseline Characteristics of Community Residents

Eight hundred and seventy-three community residents (517 from the Wujing community and 356 from the Maqiao community) agreed to take part in a face-to-face interview. The response rate is 97.76%, with 20 residents excluded (four residents refused to participate, eight residents had difficulty communicating with community physicians, and eight residents with other diagnoses). No missing information was found among all the participants. The mean age of all community residents was 65.69 years (standard deviation 10.2, Table 1). The prevalence of hypertension, diabetes, previous stroke, and cancer of any type was 68.73, 29.01, 12.17, and 3.32%, respectively. The percentage of smokers in residents living in the Wujing community was higher than for those living in the Maqiao community (36.94 vs. 28.45%, *P* = 0.009). The number of residents with hypertension was larger in the Maqiao community in comparison to the Wujing community (80.9 vs. 60.35%, *P* < 0.001). It is worth noting the prevalence of prior stroke was almost six times higher for residents living in the Maqiao community than for residents living in the Wujing community (24.01 vs. 4.06%, *P* < 0.001). More residents had a past medical history of cancer in the Wujing community than in the Maqiao community (4.84% vs. 1.12%, *P* = 0.003).

**Table 1:** Baseline characteristics of community residents.

		**Whole group**	**Wujing community**	**Maqiao community**	
		***n* = 873**	***n* = 517**	***n* = 356**	***P***
Age		65.69 (10.2)	65.79 (10.15)	65.53 (10.27)	0.623
Gender	Male	448 (51.32%)	265 (51.26%)	183 (51.40%)	0.966
	Female	425 (48.68%)	252 (48.74%)	173 (48.60%)	
Drinking habit		202 (23.14%)	123 (23.79%)	79 (22.19%)	0.582
Smoking habit		292 (33.49%)	191 (36.94%)	101 (28.45%)	**0.009**
Past medical history					
Hypertension		600 (68.73%)	312 (60.35%)	288 (80.90%)	**<0.001**
Diabetes		253 (29.01%)	146 (28.24%)	107(30.14%)	0.543
Previous stroke		106 (12.17%)	21 (4.06%)	85 (24.01%)	**<0.001**
Cancer of any type		29 (3.32%)	25 (4.84%)	4 (1.12%)	**0.003**

*Data were shown as mean (standard deviation) or number (percentage). P value stands for the differences between the two communities. Bold values are statistically significantly*.

### The Prevalence of Suspected TIA During the Covid-19 Pandemic

A total of 143 (16.38%) suspected TIA cases were identified from the 873 community residents living in the suburb of Shanghai (89 of them from the Wujing community and 54 from the Maqiao community respectively). As for each subtype of the suspected TIA, 8.39% displayed only motor weakness, 1.4% of them had only aphasia, 25.17% of them suffered only from a visual-field defect, and 31.47% of them had only with the sensory deficit. There were 33.57% of all the suspected TIA cases with more than one typical symptom. Factors associated with the suspected TIA of the 873 community residents in the univariate analysis were older age (mean age 67.95 vs. 65.25, *P* = 0.007), higher prevalence of previous stroke (27.97 vs. 9.07%, *P* < 0.001), and cancer (6.99 vs. 2.6%, *P* = 0.007) (Supplementary Table 1).

### The Behavior of Community Residents With Suspected TIA

**Figure 1:**
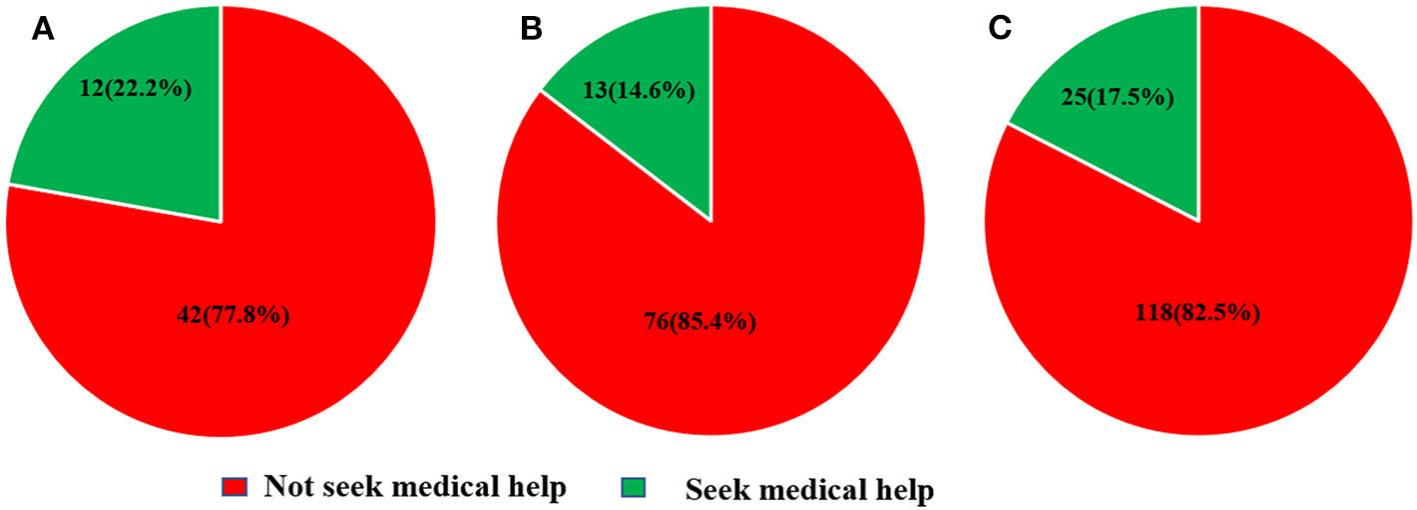
The behavior of community residents with suspected transient ischemic attack. **(A)** Residents from Maqiao community. **(B)** Residents from Wujing community. **(C)** All residents.

**Figure 2:**
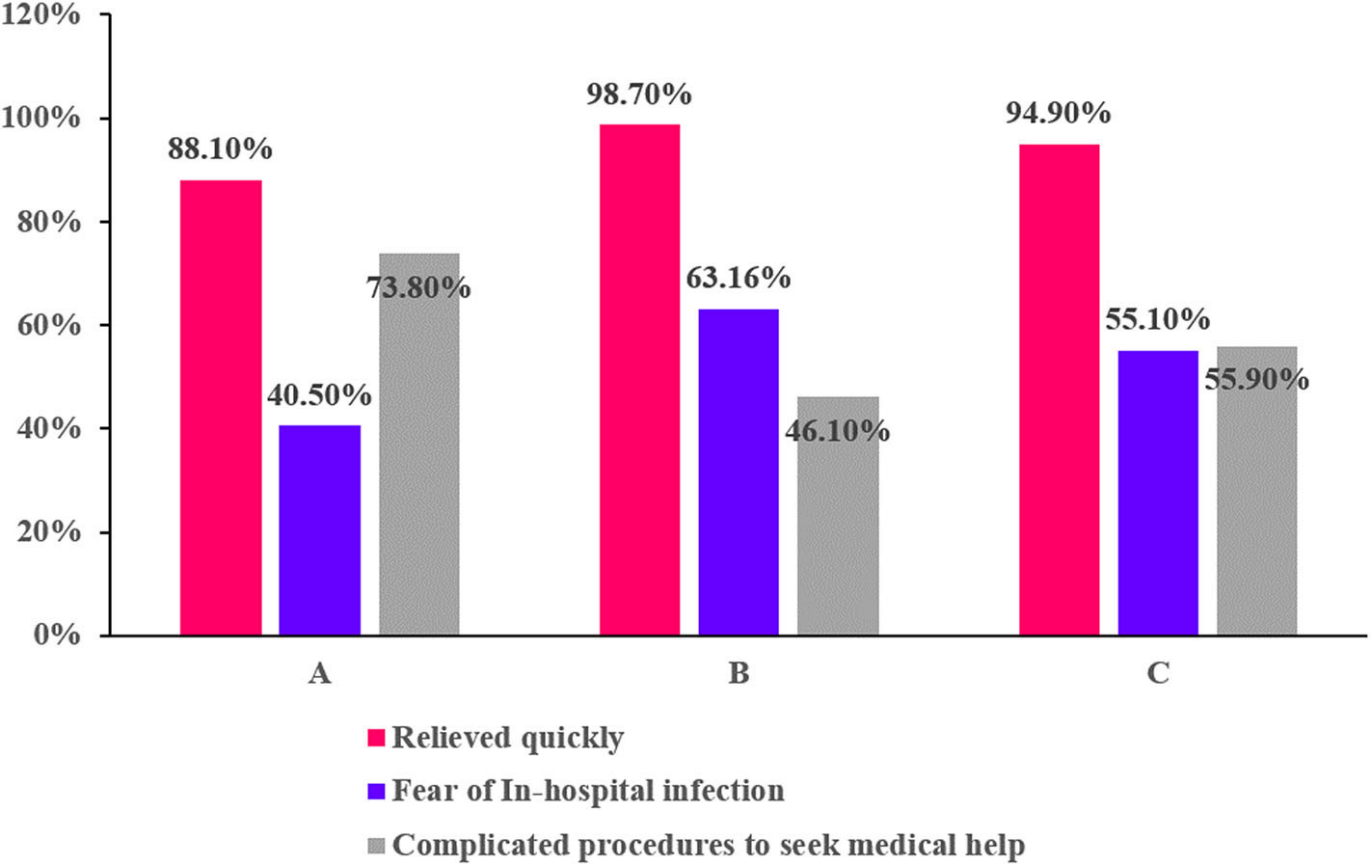
Reasons for not seeking medical care. **(A)** Residents from the Maqiao community. **(B)** Residents from the Wujing community. **(C)** All residents.

Figure 1 indicates that <20% of the community residents with suspected TIA went to the hospital during the Covid-19 pandemic. The number of residents with suspected TIA who sought medical attention in the Maqiao community was 12 (22.2%) and 13 (14.6%) in the Wujing community respectively (Figure 1). Of all 25 residents who sought medical attention, four were diagnosed with high-risk TIA and prescribed dual antiplatelet therapy. Ten were diagnosed with TIA and prescribed antiplatelet therapy (aspirin or clopidogrel). All of them received a clinical assessment from physicians of the emergency department or neurology department. The most common reason for not seeking medical attention during the pandemic was still that the symptom relieved quickly (94.9%) (Figure 2). A total of 63.16% of the residents with suspected TIA living in the Wujing community avoided going to the hospital out of the consideration of in-hospital infection, while for residents living in the Maqiao community, 73.8% of them pointed out that the complicated procedures of seeking medical care during the pandemic made them hesitate to go to the hospital. Of all the residents, only 9 (1.03%) knew TIA. Table 2 shows a multivariate analysis of factors associated with seeking medical attention during the pandemic. Female residents with suspected TIA were less likely to seek medical attention in comparison with male residents (odds ratio 0.25, 95% Confidence Interval 0.07–0.88, *P* = 0.03). Residents with the previous history of stroke tended to go to the hospital when they had suspected TIA (odds ratio 2.89, 95% Confidence interval 1.07–7.84, *P* = 0.037). As for the manifestation of the suspected TIA, residents with aphasia or dysarthria were more likely to seek medical help (odds ratio 4.25, 95% Confidence interval 1.12–16.08, *P* = 0.033). “Dual” suspected TIA within 1 week were also found to be strongly associated with seeking medical attention among the community residents (odds ratio 6.42, 95% Confidence interval 2.23–18.52, *P* = 0.001). As for the stroke risk prediction score, ABCD3, but not ABCD2, was significantly associated with seeking medical attention.

**Table 2:** Factors associated with seeking medical attention during the Covid-19 pandemic.

	**Univariate analysis**	***P***	**Multivariate analysis**	***P***
	**OR (95% CI)**		**OR (95% CI)**	
Age	0.98 (0.93, 1.03)	0.407	0.95 (0.9, 1.01)	0.076
Gender (Female vs. Male)	0.56 (0.22, 1.39)	0.211	0.25 (0.07, 0.88)	**0.03**
Drinking habit	0.75 (0.28, 2.03)	0.571	0.35 (0.1, 1.25)	0.105
Smoking habit	0.65 (0.25, 1.69)	0.38	0.34 (0.1, 1.11)	0.074
Past medical history
Hypertension	1.13 (0.41, 3.08)	0.814	1.57 (0.49, 5.05)	0.449
Diabetes	0.56 (0.21, 1.52)	0.258	0.41 (0.14, 1.25)	0.119
Previous stroke	2.97 (1.22, 7.24)	**0.017**	2.89 (1.07, 7.84)	**0.037**
Cancer of any type	1.2 (0.24, 0.6)	0.828	1.57 (0.27, 9.11)	0.62
Manifestation of the suspected TIA
BP higher than 140/90 mmHg	1.39 (0.46, 4.18)	0.56	1.3 (0.38, 4.44)	0.68
Motor weakness in two limbs or in one limb and the face	1.53 (0.64, 3.68)	0.34	0.94 (0.33, 2.68)	0.91
Aphasia or dysarthria	6.17 (1.93, 19.67)	**0.002**	4.25 (1.12, 16.08)	**0.033**
Visual-field defect or monocular blindness	1.82 (0.76, 4.34)	0.18	2.71 (0.97, 7.61)	0.058
Sensory deficit in two limbs or in one limb and the face	1.14 (0.46, 2.78)	0.781	0.87 (0.31, 2.45)	0.79
Two or more of the above symptoms	2.57 (1.07, 6.18)	**0.035**	1.7 (0.61, 4.75)	0.312
Dual attack within 1 week	6.61 (2.61, 16.73)	**<0.001**	6.42 (2.23, 18.52)	**0.001**
Duration of the symptom (more than 1 h)	7.91 (1.25, 50.12)	**0.028**	7.24 (0.99, 53.08)	0.052
The stroke risk of the suspected TIA
ABCD2	1.19 (0.91, 1.56)	0.213	1.08 (0.78, 1.51)	0.642
ABCD3	1.36 (1.01, 1.68)	**0.005**	1.35 (1.03, 1.78)	**0.03**

*BP, blood pressure; TIA, transient ischemic attack; OR, odds ratio; CI, confidence interval; Age, ABCD2, and ABCD3 as continuous variable; Multivariate analysis adjusted for age, gender, drinking habits, smoking habits, hypertension, diabetes, previous stroke, and cancer of any type. Bold values are statistically significantly*.

## Discussion

To our knowledge, this is the first study to explore the impact of the Covid-19 pandemic on the behavior of residents living in the community seeking medical attention after TIA. Our study indicates that more than half of community residents with suspected TIA did not go to the hospital during the pandemic. The major reason for this was still that symptoms disappeared quickly, though the pandemic did have an impact on the behavior of those seeking medical attention. When we combined the Covid-19-related causes, 5.08% of all the suspected TIA cases chose Covid-19-related arguments as their sole reasons for not seeking medical care, while 74.58% of them chose Covid-19-related arguments and the quick disappearance of symptoms together as the reasons for not seeking medical care. The decision not to seek medical attention after a suspected TIA is likely to be made for multiple reasons, especially under the circumstances of the pandemic. Of all the suspected TIA cases, only those with dual attack within 1 week or aphasia (or dysarthria) were strongly associated with seeking medical attention during the pandemic. Since the stroke risk after TIA without timely medical care was huge, the outcome for those community residents with suspected TIA who chose to stay at home was potentially devastating (10).

Several hospital-based stroke registry data indicated that the number of admissions for stroke, TIA, or minor stroke decreased significantly during the Covid-19 pandemic. A study in Hong Kong compared the stroke registry data of a comprehensive stroke center 60 days after the first diagnosis of Covid-19 case with the same period in 2019 and noticed a nearly 80% reduction in the presentation with TIA (4). The drop in the presentation with TIA was also observed in Brazil, Germany, and Norway, ranging from 30 to 80% (3, 5, 6).

Following the outbreak of Covid-19 in the Chinese mainland, strict official policies from the centers for disease control and prevention have been released to prevent hospital-related infection of Covid-19. Community residents who want to seek medical attention during the pandemic, first of all, need to make an online appointment with the doctor on the hospital's website or application (11). All patients are required to take their body temperature and finish an online survey regarding travel history and any potential exposure to Covid-19 when they arrive at the hospital. A chest CT scan, blood tests, and nucleic acid testing for Covid-19 were also mandatory examinations for patients admitted to the hospital. As we have shown in the results, we observed that in addition to the impact on the behavior of those seeking medical care of the fear of in-hospital infection with Covid-19, the complicated nature of procedures for seeking medical care during the pandemic also hindered community residents from going to the hospital.

Public stroke education was recommended to be continued during the pandemic to improve the behavior of residents with stroke or TIA, while a study from the United Kingdom indicated that extensive FAST-based public education failed to improve the residents' response to TIA and minor stroke even without the impact of the pandemic (12). Public stroke education program tailored to minor stroke and TIA is urgently needed in the future. In the context of the Covid-19 pandemic, telemedicine seems to be a promising way to support the treatment of TIA (13). It allows communication between community residents and stroke physicians and at the same time avoids interaction with other people. Telemedicine has also shown its potential in delivering guided behavioral therapy for post-stroke anxiety (14).

### Limitations

We have to admit that there are several limitations of this study. First, this is not a population-based study, and the residents were all living in the suburbs of Shanghai, resulting in differences in the prevalence of TIA in comparison with a population-based study published in 2015, which revealed that the prevalence of suspected TIA was 8.68% (15). The education level of community residents was lower in comparison with residents living in the city, which may lead to the discrepancy of seeking medical attention after TIA. Second, in addition to the three predefined potential reasons for not seeking medical care, there might be other reasons, such as living alone or the negative impact of the stay-at-home order. However, they were not the focus of this study. Third, in consideration of errors in recollection of the community residents, we conducted only one-month-and-a-half face-to-face interview, which may lead to less representative samples of the residents living in the two communities. Since the participants in our study are seeking management of chronic diseases when they arrive at the community health service center, older age and higher prevalence of vascular risk factors may also limit the generalizability of the conclusion. Last, the diagnosis of TIA can sometimes be challenging even for experienced stroke physicians. Although we conducted an online stroke education for the two community physicians, those identified suspected TIA cases were not adjudicated or verified by a stroke physician, leading to potential misdiagnosis of the patient. As indicated in a population-based study including 98,658 participants from 31 provinces across mainland China, the prevalence of suspected TIA identified by trained staff was 8560/98658 (8.68%), while it decreased to 2780/98658 (2.82%) after being verified by neurologists (15). The over-diagnosis of suspected TIA may lead to a lower rate of patients with suspected TIA during the pandemic seeking medical attention.

## Conclusion

Our study indicates that <20% of the community residents with suspected TIA went to the hospital during the Covid-19 pandemic. The most common reason for not going to the hospital was still that the symptoms disappeared quickly; however, the pandemic did have an impact on the behavior of seeking medical attention. Fear of in-hospital infection and complicated procedures involved in seeking medical attention during the pandemic contributed to the failure to seek medical attention after the suspected TIA. Residents with a dual attack within 1 week or with aphasia or dysarthria were more likely to seek medical attention during the pandemic.

## Data Availability Statement

The raw data supporting the conclusions of this article will be made available by the authors, without undue reservation.

## Ethics Statement

These studies involving human participants were reviewed and approved by Institutional Review Board of Wujing community health service center and Maqiao community health service center. The patients/participants provided their written informed consent to participate in this study. Written informed consent was obtained from the individual(s) for the publication of any potentially identifiable images or data included in this article.

## Author Contributions

SY and BL: study concept, acquisition of data, analysis and interpretation of data, and drafting of the manuscript. YLi and YLu: analysis and interpretation of data, drafting of the manuscript. QX: acquisition of data, analysis and interpretation of data. JH: analysis and interpretation of data. ZY: study design, study supervision or coordination, and revising the manuscript. XL: study concept and design, study supervision or coordination, and revising the manuscript. All authors contributed to the article and approved the submitted version.

## Conflict of Interest

The authors declare that the research was conducted in the absence of any commercial or financial relationships that could be construed as a potential conflict of interest.

## References

[1] Markus HS Brainin M. COVID-19 and stroke-A global World Stroke Organization perspective. Int J Stroke. (2020) 15:361–4. 10.1177/1747493020923472 32310017 PMC11927026

[2] Mahase E. Covid-19: WHO declares pandemic because of alarming levels of spread, severity, and inaction. BMJ. (2020) 368:m1036. 10.1136/bmj.m1036 32165426

[3] Kristoffersen ES Jahr SH Thommessen B Ronning OM. Effect of Covid-19 pandemic on stroke admission rates in a Norwegian population. Acta Neurol Scand. (2020). 10.1111/ane.13307. [Epub ahead of print]. 32620027 PMC7361547

[4] Teo KC Leung WCY Wong YK Liu RKC Chan AHY Choi OMY. Delays in stroke onset to hospital arrival time during COVID-19. Stroke. (2020) 51:2228–31. 10.1161/STROKEAHA.120.030105 32432998 PMC7258759

[5] Hoyer C Ebert A Huttner HB Puetz V Kallmunzer B Barlinn K. Acute stroke in times of the COVID-19 pandemic: a multicenter study. Stroke. (2020) 51:2224–7. 10.1161/STROKEAHA.120.030395 32516064

[6] Diegoli H Magalhaes PSC Martins SCO Moro CHC Franca PHC Safanelli J. Decrease in hospital admissions for transient ischemic attack, mild, and moderate stroke during the COVID-19 era. Stroke. (2020) 51:2315–21. 10.1161/STROKEAHA.120.030481 32530738 PMC7302100

[7] Montaner J Barragan-Prieto A Perez-Sanchez S Escudero-Martinez I Moniche F Sanchez-Miura JA. Break in the stroke chain of survival due to COVID-19. Stroke. (2020) 51:2307–14. 10.1161/STROKEAHA.120.030106 32466738 PMC7282408

[8] Amarenco P Transient ischemic attack. N Engl J Med. (2020) 382:1933–41. 10.1056/NEJMcp1908837 32402163

[9] Kiyohara T Kamouchi M Kumai Y Ninomiya T Hata J Yoshimura S. ABCD3D and ABCD3D-I scores are superior to ABCD2 score in the prediction of short- and long-term risks of stroke after transient ischemic attack. Stroke. (2014) 45:418–25. 10.1161/STROKEAHA.113.003077 24335223

[10] Coull AJ Lovett JK Rothwell PM Oxford Vascular S. Population based study of early risk of stroke after transient ischaemic attack or minor stroke: implications for public education and organisation of services. BMJ. (2004) 328:326. 10.1136/bmj.37991.635266.44 14744823 PMC338101

[11] Wang X Chen Y Li Z Wang D Wang Y. Providing uninterrupted care during COVID-19 pandemic: experience from Beijing Tiantan Hospital. Stroke Vasc Neurol. (2020) 5:180–4. 10.1136/svn-20200-000400 32385131 PMC7246102

[12] Wolters FJ Li L Gutnikov SA Mehta Z Rothwell PM. Medical attention seeking after transient ischemic attack and minor stroke before and after the UK Face, Arm, Speech, Time (FAST) public education campaign: results from the Oxford vascular study. JAMA Neurol. (2018) 75:1225–33. 10.1001/jamaneurol.2018.1603 29971433 PMC6233848

[13] Klein BC Busis NA. COVID-19 is catalyzing the adoption of teleneurology. Neurology. (2020) 94:903–4. 10.1212/WNL.0000000000009494 32238505

[14] Chun HY Carson AJ Tsanas A Dennis MS Mead GE Calabria C. Telemedicine cognitive behavioral therapy for anxiety after stroke: proof-of-concept randomized controlled trial. Stroke. (2020) 51:2297–306. 10.1161/STROKEAHA.120.029042 32576090 PMC7382539

[15] Wang Y Zhao X Jiang Y Li H Wang L Johnston SC. Prevalence, knowledge, and treatment of transient ischemic attacks in China. Neurology. (2015) 84:2354–61. 10.1212/WNL.0000000000001665 25957333 PMC4464739

