# Supplementary material for: Impact of Covid-19 on the Behavior of Community Residents With Suspected Transient Ischemic Attack
Source: Front Neurol. 2020 Oct 16;11:590406. doi: 10.3389/fneur.2020.590406 (PMC7596267; doi:10.3389/fneur.2020.590406)
Supplement: Supplementary file 1 [file Table_1.DOCX]

**Supplement material**

**Community-based survey for the behavior of seeking medical attention after transient ischemic attack during the Covid-19 pandemic**

1. How old are you?
2. Your gender?
3. Are you a smoker?

Yes, I am. (include those used to be a smoker)

No, I’m not.

1. Are you a drinker?

Yes, I am. (include those used to be a drinker)

No, I’m not.

1. Past medical history (1 for yes, 0 for no)

Hypertension?

Diabetes?

Hyperlipidemia?

Cancer of any type?

Previous stroke event?

(The diagnosis was defined according to standard definitions and with medical record from local tertiary hospital)

1. If you are a patient with hypertension and it controlled not well during the pandemic, was your blood pressure higher than 140/90mmhg (even for once)? (1 for yes, 0 for no)
2. Did you experience any of the symptoms listed below which lasted for only minutes and relieved quickly during the pandemic? (1 for yes, 0 for no) (multichoice)
3. Motor weakness in two limbs or in one limb and the face?
4. Aphasia or dysarthria?
5. Visual-field defect (homonymous hemianopia) or monocular blindness?
6. Sensory deficit in two limbs or in one limb and the face?
7. If you experienced any of the symptoms, did they occur again within one week? (1 for yes, 0 for no)
8. Is the blood pressure higher than 140/90mmhg at the onset of transient ischemic attack?
9. Did the symptoms you experienced last more than 60 minutes? (1 for yes, 0 for no)
10. Did you seek the medical attention immediately after the symptom onset? (1 for yes, 0 for no)
11. Out of what reasons made you decide not to seek medical attention immediately during the pandemic? (1 for yes, 0 for no) (multichoice)
12. The symptoms relieved quickly.
13. Fear of in-hospital infection of the Covid-19.
14. The procedures you need to complete to seek medical help during the pandemic were quite complicated. In other words, the complicated procedures hindered you from presenting to hospital.
15. Have you heard about the disease called transient ischemic attack? (1 for yes, 0 for no)

Yes means that the resident can name at least one of the typical symptoms and know it is related to ischemic stroke.

1. The medication history for the suspected TIA who sought medical attention during the pandemic?

Table 1 Univariate analysis of the factors associated with suspected TIA

|  |  | Without suspected TIA | With suspected TIA |  |
| --- | --- | --- | --- | --- |
|  |  | N=730 | N=143 | P |
| Age |  | 65.25(10.32) | 67.95(9.24) | **0.007** |
| Gender | male | 367(50.27%) | 81(56.64%) | 0.163 |
|  | female | 363(49.73%) | 62(43.36%) |  |
| Drinking habit | | 161(22.05%) | 41(28.67%) | 0.086 |
| Smoking habit | | 241(33.06%) | 51(35.66%) | 0.546 |
| Past medical history | |  |  |  |
| Hypertension | | 494(67.67%) | 106(74.13%) | 0.128 |
| Diabetes |  | 205(28.08%) | 48(33.80%) | 0.169 |
| Previous stroke | | 66(9.07%) | 40(27.97%) | **<0.001** |
| Cancer of any type | | 19(2.6%) | 10(6.99%) | **0.007** |

Data were shown as number (percentage) or mean (standard deviation), TIA transient ischemic attack
